# GIDB: a knowledge database for the automated curation and multidimensional analysis of molecular signatures in gastrointestinal cancer

**DOI:** 10.1093/database/baz051

**Published:** 2019-05-15

**Authors:** Ying Wang, Yueqian Wang, Shuangkuai Wang, Yuantao Tong, Ling Jin, Hui Zong, Rongbin Zheng, Jinxuan Yang, Zeyu Zhang, En Ouyang, Mengyan Zhou, Xiaoyan Zhang

**Affiliations:** 1 Research Center for Translational Medicine, Shanghai East Hospital, School of Life Sciences and Technology, Tongji University, Shanghai, China; 2 Department of Laboratory Medicine, Shanghai Eastern Hepatobiliary Surgery Hospital, Shanghai, China

## Abstract

Gastrointestinal (GI) cancer is common, characterized by high mortality, and includes oesophagus, gastric, liver, bile duct, pancreas, rectal and colon cancers. The insufficient specificity and sensitivity of biomarkers is still a key clinical hindrance for GI cancer diagnosis and successful treatment. The emergence of `precision medicine’, `basket trial’ and `field cancerization’ concepts calls for an urgent need and importance for the understanding of how organ system cancers occur at the molecular levels. Knowledge from both the literature and data available in public databases is informative in elucidating the molecular alterations underlying GI cancer. Currently, most available cancer databases have not offered a comprehensive discovery of gene-disease associations, molecular alterations and clinical information by integrated text mining and data mining in GI cancer. We develop GIDB, a panoptic knowledge database that attempts to automate the curation of molecular signatures using natural language processing approaches and multidimensional analyses. GIDB covers information on 8730 genes with both literature and data supporting evidence, 248 miRNAs, 58 lncRNAs, 320 copy number variations, 49 fusion genes and 2381 semantic networks. It presents a comprehensive database, not only in parallelizing supporting evidence and data integration for signatures associated with GI cancer but also in providing the timeline feature of major molecular discoveries. It highlights the most comprehensive overview, research hotspots and the development of historical knowledge of genes in GI cancer. Furthermore, GIDB characterizes genomic abnormalities in multilevel analysis, including simple somatic mutations, gene expression, DNA methylation and prognosis. GIDB offers a user-friendly interface and two customizable online tools (Heatmap and Network) for experimental researchers and clinicians to explore data and help them shorten the learning curve and broaden the scope of knowledge. More importantly, GIDB is an ongoing research project that will continue to be updated and improve the automated method for reducing manual work.

## Introduction

Gastrointestinal (GI) cancers, including oesophageal, gastric, liver, bile duct, pancreatic and colorectal cancers, are one of the major causes leading to death and have the second highest incidence rates of malignancies worldwide (1–6). Among all organ system cancers, GI cancers are responsible for more deaths from cancer than any other cancers. The burden of GI cancers has been rising, and an obviously increasing incidence trend in young adults has been observed (7, 8). However, early stage diagnosis of GI cancers remains difficult and challenging. For instance, carcinoembryonic antigen and carbohydrate antigen 19-9 (CA19-9) are the most commonly used traditional tumour biomarkers, but their levels are not recommended in screening for early detection of GI cancers as the sensitivity and specificity are both very low (9–13). The prognoses of GI cancers remain dismal. Although potential therapeutic agents have been approved for colorectal, gastric, liver and pancreatic cancers, the treatment effectiveness is often limited due to tumour and patient heterogeneity. Therefore, there is an urgent need to require a deep understanding of GI cancers for identifying predictive and prognostic and potential molecular biomarkers (14). Considering the field cancerization and the fundamental peculiarity and similarity of GI cancers in biological function, pathogenesis and potential therapeutic strategies, a knowledge of how a specific organ digestive system cancer occurs at the cellular and molecular levels is significant and meaningful (15–17).

**Figure 1:**
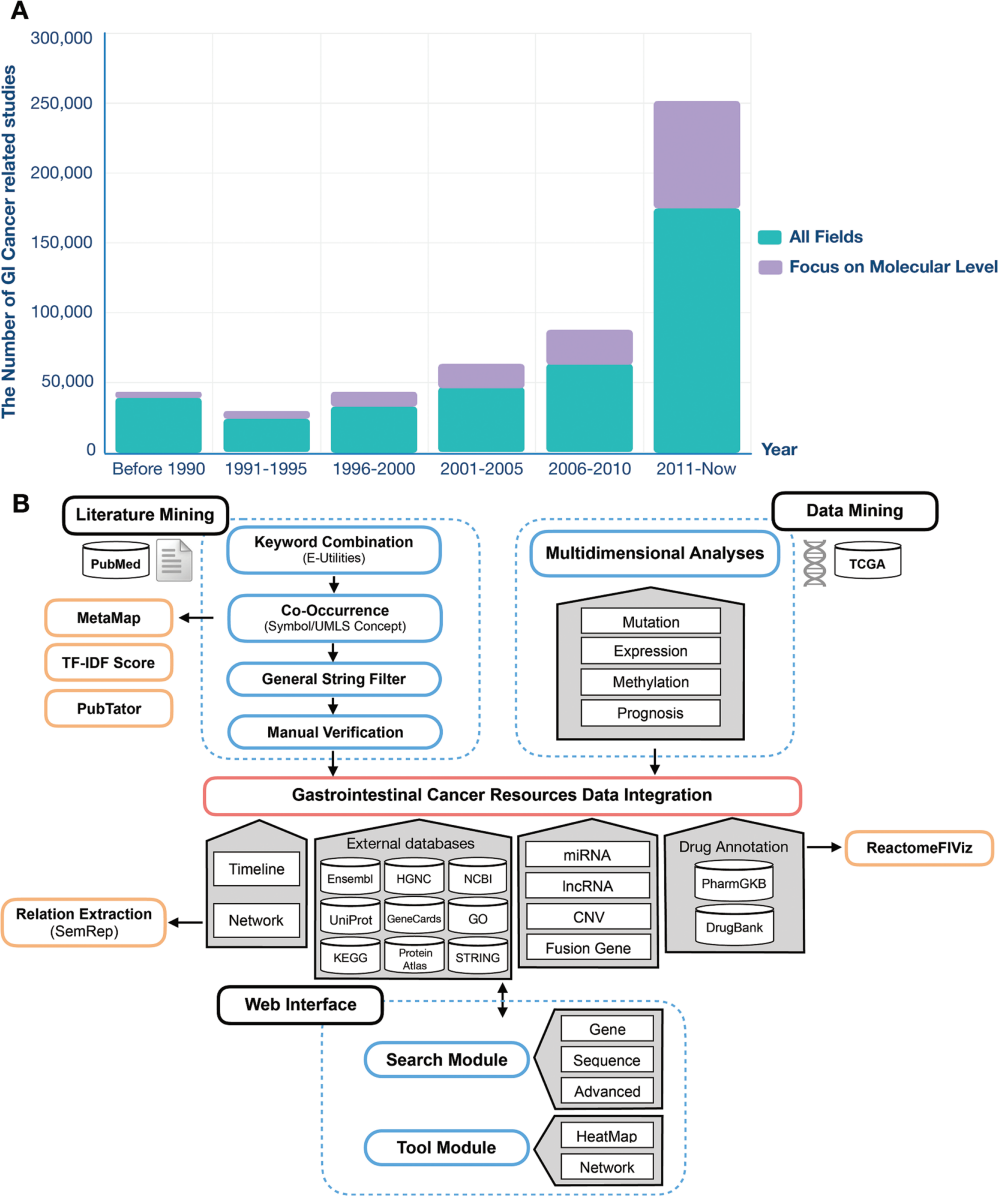
The GI cancer-related studies found in the PubMed database and the schema of the GIDB. (**A**) PubMed queries for GI cancer-related studies and GI cancer-related studies at the molecular level. (**B**) It describes the information retrieval steps used in GIDB extracted from the literature and datasets. It also shows the approaches for literature mining and data mining integrated into GIDB.

At present, a tremendous increase in the resources available in public sources has been accumulated, not only in data but also in the literature. Figure 1A shows the number of articles in PubMed related to GI cancer. The volume of data and the literature is expected to continue to grow annually, so that manual work is unable to keep up with the speed of information accumulation. Moreover, the underlying knowledge of these resources is not fully developed due to basic researchers and clinicians having limited background knowledge. Moreover, data isolation and heterogeneity cause difficulty for scientists in performing effective data mining, extraction and integration from public sources for their own research (18). There are several types of specialized databases, including CoReCG (19), DBGC (20), Liverome (21) and PED (22), which provide single-phenotype information for colorectal, gastric, liver and pancreatic cancers. Few studies have conducted text mining in data integration. A gastrointestinal cancer database (23) has been developed to provide a comprehensive molecular oncogenomic profiling of GI cancers. However, the accumulated data volume is small (463 genes and 713 miRNAs), and the data content is simple. In addition, there are also several databases and platforms for exploring pan-cancers, such as OMIM (24), COSMIC (25), HGMD (26), Oncomine™ (27) and cBioPortal (28). With respect to data content, OMIM, COSMIC and HGMD have a limited number of data types. Oncomine and cBioPortal are specialized in providing information to show links between datasets and articles. Meanwhile, for the interface, experimental researchers and clinicians may have a long learning curve.

In accordance with the above point of view, all the databases currently available have not offered a comprehensive discovery of gene-tumour associations by integrated text mining of the literature, data mining of multidimensional data and clinical information on GI cancers. Thus, a comprehensive and accessible public knowledge database in GI cancers is required to maximize the benefits of literature–data integration, which is crucial for a better understanding of the molecular events underlying GI cancers and the identification of candidate molecular markers for diagnosis and treatment.

Overall, this study involves the creation of a highly curated database, GIDB, which holds a comprehensive view of about 8700 molecular signatures mined from over 130 000 texts and 1800 samples. GIDB provides a wide range of resources for GI cancers, such as genes, miRNAs, lncRNAs, fusion genes, copy number variations (CNVs), gene-tumour semantic networks, historical `Timelines’ of GI cancer-related genes and information on cancer drugs, as well as alterations at the molecular level (somatic mutation, expression and methylation) and their impact on prognosis. Furthermore, an important aspect of the GIDB is that it is a well-designed database and has its own web analytics tools (HeatMap and Interactive Network). The GIDB will be of great benefit and convenience to experimental researchers, clinicians and bioinformatics scientists in three common situations: (i) a brief historical review of GI cancer-related genes; (ii) evidence supporting predictions; and (iii) extensive identification and in-depth experimental validation.

## Construction and content

To date, the GIDB has been populated with information from vast amounts of biomedical literature and multidimensional omics data. Both aspects are of crucial importance to show a more comprehensive view of GI cancer researches to determine the association of these findings and clinical translational applications (e.g. biomarkers). One key feature of the GIDB is that it allows the users to trace the `Cancer-Timeline’ of gene searches and the `Gene-Timeline’ of identified genes in each type of GI cancer. More importantly, the GIDB demonstrates genomic alterations and prognoses of genes and gene-disease association networks. The schema of the GIDB is represented in Figure 1B.

### Data curation

#### Semantic association for entity recognition

**Figure 2:**
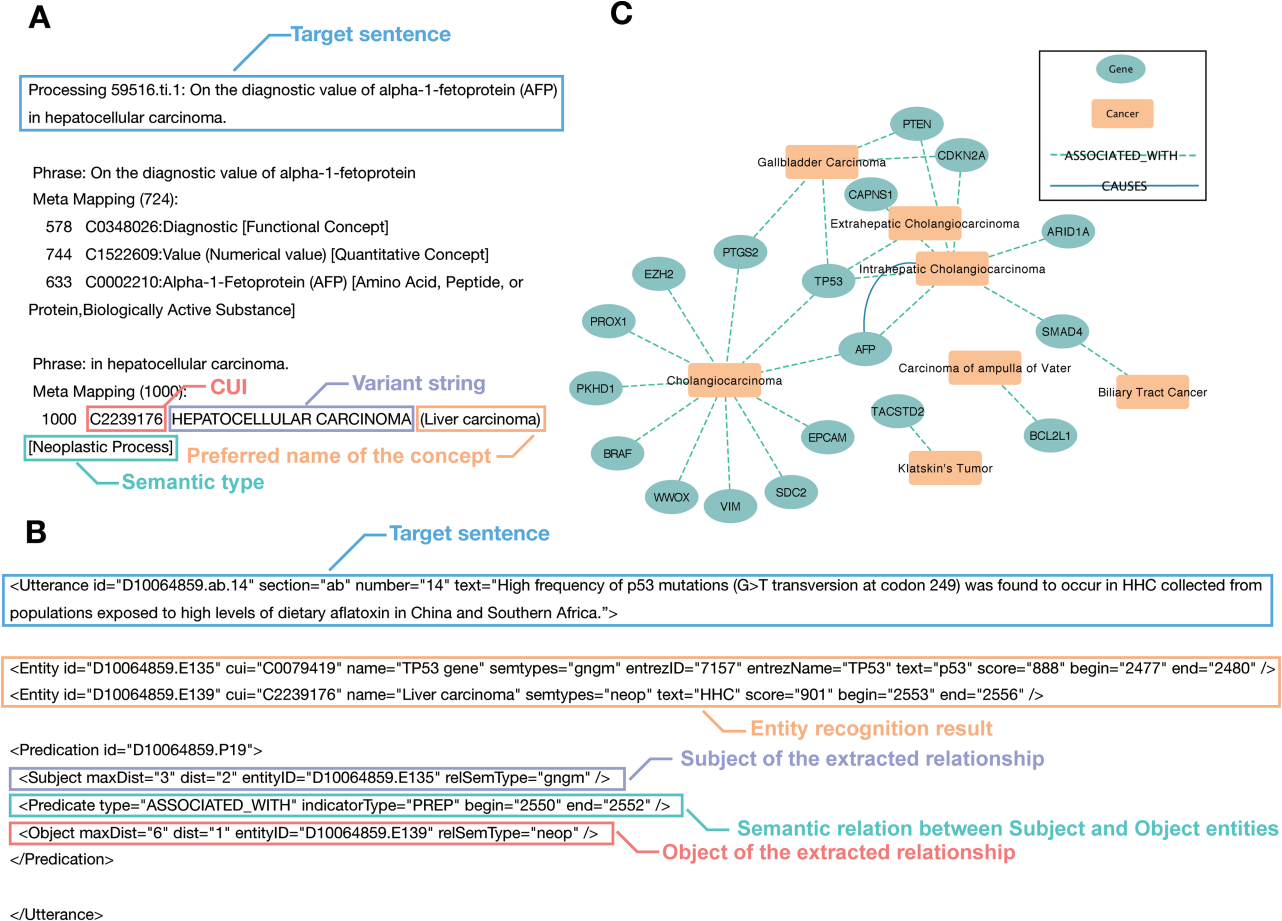
Cases of semantic association for entity recognition and semantic network inference. (**A**) A case of the MetaMap entity recognition result: the blue label represents the target sentence in the citation. The red label gives the Concept Unique Identifies in UMLS Metathesaurus. The purple label shows the variant string in each sentence, including its acronyms, abbreviations and synonyms. The orange label describes the preferred name of the concept. The green label is the semantic type of the recognized concept. (**B**) A case of the SemRep relationship extraction result: the blue label shows the target sentence in the citation. The orange label gives the entity recognition result mapping by MetaMap. The purple label and red label show the Subject and Object of the extracted relationship, respectively. The green label represents the semantic relation between subject and object entities. In this case, it means the subject `TP53 gene’ is associated with the object `Liver carcinoma’. (**C**) The semantic network in BTC: the green node represents curated genes. The orange node means the different tumour subtypes of BTC. The colour of the edge shows the SemRep extracted relation types.

In the GIDB, we perform a natural language processing (NLP) approach to automate the extraction of disease–gene association from biomedical literature in PubMed. To extract information from the semi-structured MEDLINE format, we construct relevant vocabularies for GI cancers, including cholangiocarcinoma, gallbladder carcinoma, vater ampulla carcinoma, hepatocellular carcinoma, gastric cancer, pancreatic cancer, oesophageal cancer, colon cancer and rectal cancer. The metathesaurus concepts and semantic types mentioned in the text are recognized by MetaMap (Figure 2A). Overall, it corresponds to 20 630 Unified Medical Language System (UMLS) concepts, including 20 595 gene concepts and 35 tumour concepts.

#### Semantic network inference

The semantic associations of entities in text are identified by SemRep in the form of a semantic knowledge network. The GIDB currently accommodates semantic relations on AFFECTS, ASSOCIATED_WITH, AUGMENTS, CAUSES, DISRUPTS, PREVENTS and TREATS (Figure 2B). One can view how genes (nodes) interact (edges) in each GI cancer type (nodes) in a 3D network mode. There are presently 2381 unique interactions in the GIDB (176 for bile duct cancer, 977 for colorectal cancer, 197 for oesophageal cancer, 530 for liver cancer, 120 for pancreatic cancer and 381 for gastric cancer). More importantly, prediction of semantic network subgroups, including intrahepatic cholangiocarcinoma, Klatskin’s tumour, gallbladder carcinoma, extrahepatic cholangiocarcinoma and carcinoma of ampulla of vater in biliary tract cancer (BTC) is shown in Figure 2C.

### Evaluating

With respect to the database content in the GIDB, an important aspect to consider is the automatic extraction of signatures from biomedical literature, which needs to be addressed comprehensively and unambiguously. To estimate our text-mining method, we randomly selected 790 manually curated studies from the citation collections. We observe that the true positive (TP) studies limited to positive evidence can result in a recall of 74.75% and a precision of 60.72%. Secondly, we consider the gold standard of gene–disease association for both positive and negative evidence, which improves the recall and precision by up to 75.59% and 63.53%, respectively. Thirdly, when considering the comprehensiveness of information, uncertain evidence is also classified as positive sample sets and achieves a recall of 75.9% and a precision of 68.67%. It is important to note that the automated text-mining method may lead to both false-positive and false-negative annotations due to improper term unification. Furthermore, we observed that using gene aliases as a complement to official gene symbols dramatically elevates the rate of recall (91.11%), and the integration of text mining and data mining further increases the precision to a great extent (83.35%).

### Data content

The GIDB is an ongoing research project that will continue to be updated with a semi-automated programme running on the background server to further improve the quality of data periodically and release them to the website.

**Figure 3:**
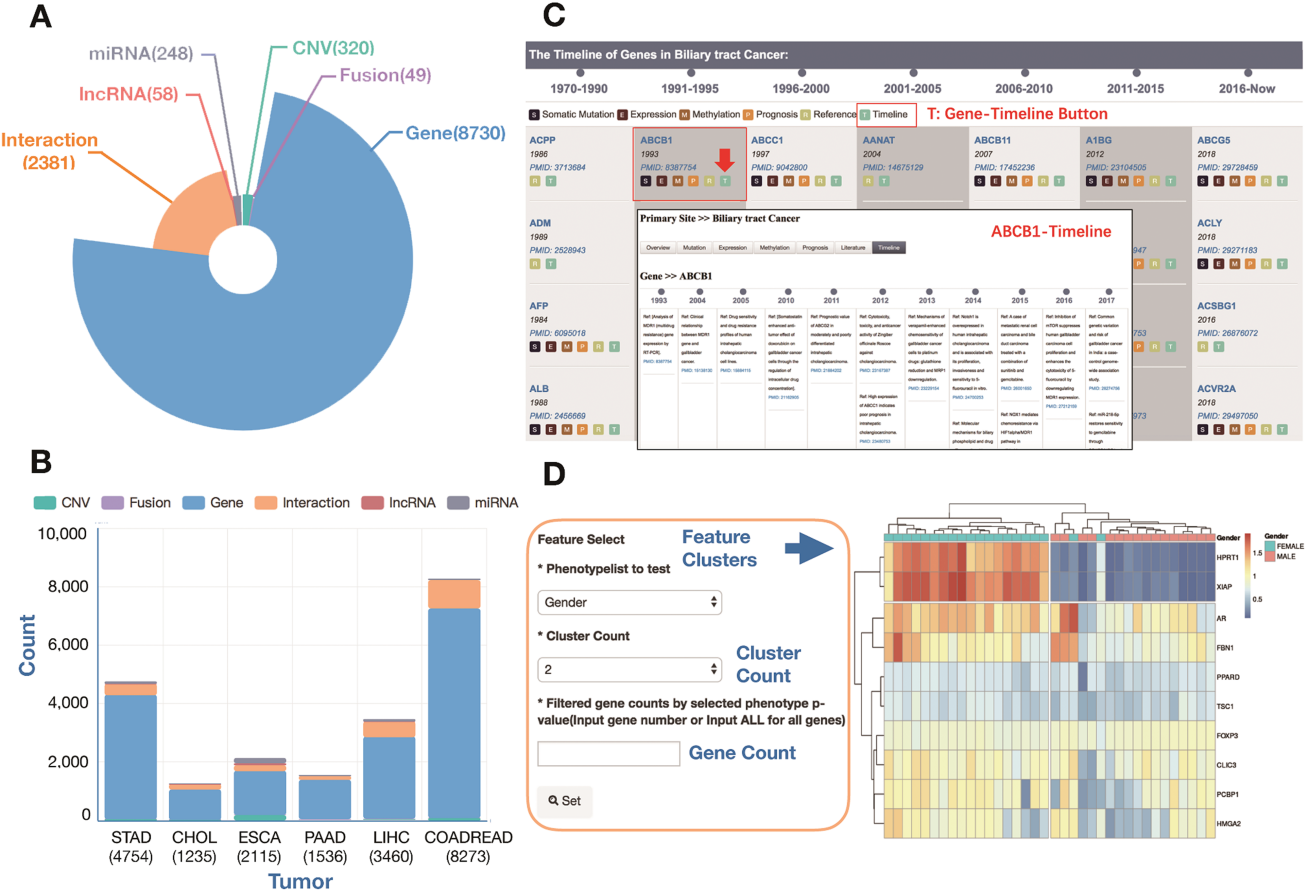
The statistics of curated signatures in the current version of the GIDB database. (**A**) A pie chart showing the number of curated signatures in GIDB, including gene, fusion gene, CNV, miRNA, lncRNA and interaction. (**B**) A histogram chart showing the number of curated signatures in each type of GI cancer. (**C**) A case of `BTC Gene-Timeline’ feature and `ABCB1 Gene-Timeline’ feature in BTC. (**D**) A case using the heatmap analysis tool: GIDB supports two types of datasets (Expression and Methylation) and two characteristics for classification (Gender and Age). The number of gene clusters or sample clusters can be adjusted manually. In the right panel, a list of selected curated genes is displayed.

The GIDB gives an instant overview of lists of curated information associated with GI cancers and provides links to detailed information on individual tumours. The GIDB consists of curated molecular signatures on protein coding genes, miRNAs-target, lncRNAs, fusion genes and CNVs. Overall, The GIDB has currently mined over 130 000 citations for biomedical literature from PubMed and 1874 samples from six GI cancer TCGA projects in which a total of 8730 genes relevant to GI cancers were found. Among them, 1023 (11.7%), 7173 (82.2%), 1503 (17.2%), 2805 (32.1%), 1353 (15.5%) and 4257 (48.8%) genes were identified in bile duct, colorectal, oesophageal, liver, pancreatic and gastric cancers, respectively. The six types of GI cancer shared 122 genes in common. Additionally, the database holds information on 248 miRNAs, 58 lncRNA, 49 fusion genes, 320 CNVs and a total of 2381 semantic networks. Figure 3A and B summarizes the distribution of signatures associated with GI cancers. Through the web pages, these signatures can be queried and displayed in tables.

### Timeline

The Timeline is an interactive and data-rich resource that provides a historical overview of major molecular discoveries in each type of GI cancer. A span of ~50 years from the 1970s to the last update time in this version of the GIDB in 2018 is chronicled in the Timeline (Figure 3C, an example of the genes in BTC). There are two kinds of `Timeline’ features, Cancer-Timeline and Gene-Timeline, highlighting the most comprehensive overview, research hotspots in each type of GI cancer and the improvement of historical knowledge of each gene across all GI cancer types. Moreover, `Timeline’ features exhibit key genes, when they were discovered and who made the discovery. We believe that many researchers in the field could benefit from it.

### Cancer heatmap and heatMap analytics tool

GIDB provides cancer heatmaps using hierarchical clustering and Euclidean distance and shows the results of curated genes in each cancer type. It displays two data types (Expression, Methylation) and several characteristics for classification (Gender, Sex, Race, AJCC_Metastasis, Family_History, Age, etc). Moreover, the GIDB developed a HeatMap analytics tool to identify distinct molecular features among subgroups with different clinicopathological characteristics. In this database, we used TCGA clinical data to distinguish the differences between normal and tumour tissues. K-means cluster analysis is conducted on gene expression data or DNA methylation data. In each primary site’s heatmap module, all curated genes’ methylation/expression datasets can be downloaded for in-depth analysis (Figure 3D, an example in BTC). Also, researchers can upload their own data and set the cut-off values using heatmap tool.

### Simple queries

A series of web pages provide an easy way to interrogate the GIDB and present information in figures and tables. Firstly, hyperlinks that lead to other web resources, such as Entrez Gene, HGNC, Ensembl, Vega, UniprotKB, Gene Cards, Gene Ontology, KEGG, ProteinAltas, STRING, PubMed, Pharmgkb and DrugBank databases, make the GIDB well-equipped for advanced searching. Secondly, the GIDB provides information on three different types of simple searches, including gene, sequence and advanced options that lets users to do sophisticated searches by combining the various concepts (genes, cancers, keywords) they have already identified for their search.

### Dictionary of cancer drugs and genes

The GIDB integrates information about curated gene sets and anticancer drugs approved by the US Food and Drug Administration using ReactomeFIViz. There are two types of links that can be used in the Gene-Cancer Drugs Dictionary page. Firstly, a gene is linked to the Gene Search Result page where users can find the detail of its relationship to GI cancer. Secondly, the information in PharmGKB and Drugs columns is related to two other online databases, the Pharmacogenetics Knowledge Base (PharmGKB) and DrugBank, to find more details for genes, drugs and interactions. Moreover, the GIDB shows a small drug–target interaction network that contains information on which genes are targeted by cancer drugs that are annotated via ReactomeFIViz. There is a total of 137 gene–drug pairs in the current database. Researchers can utilize these references and observe gene interactions with cancer drugs in networkD3 visualizations.

### Molecular alterations

A gene signature table for each GI cancer type consists of multidimensional analysis relevant to high mutation frequencies, differential expression, altered DNA methylation and survival, which describes the molecular alterations reported in GI cancers and the strength of the correlation between the genes and prognosis. Among the molecular alteration records in the GIDB, there are currently 7681 for somatic mutations, 4876 for differential expressed genes, 2102 for altered DNA methylation levels, 1797 for the impact of prognosis at the expression level (*P* < 0.05) and 1229 for the impact of prognosis at the methylation level (*P* < 0.05). Additionally, the references that are highly related to gene–disease associations display the title, journal, author lists and links to PubMed and a research overview.

### Use case

We illustrate the use of the GIDB with the example of the topoisomerase (DNA) II alpha (TOP2A) gene in BTC. Generally, we know that the DNA topoisomerase can regulate the topologic states of DNA during transcription. TOP2A is a target for anticancer agents due to its function in encoding DNA topoisomerase, and a variety of different mutations in TOP2A are correlated with the development of drug resistance (29–31).

In GIDB, we indicate that TOP2A is associated with all types of GI cancer. The evidence concerning the TOP2A gene relevant to BTC is reviewed in three references. As shown in Gene-Timeline, Washiro M *et al.* (29) first discovered this gene in 2008, and they demonstrated that highly expressed TOPO Ilalpha in gallbladder cancer might be an effective target for chemotherapy. More interestingly, the GIDB correctly identified the TOP2A protein, even though TOPO II alpha is not its official name.

**Figure 4:**
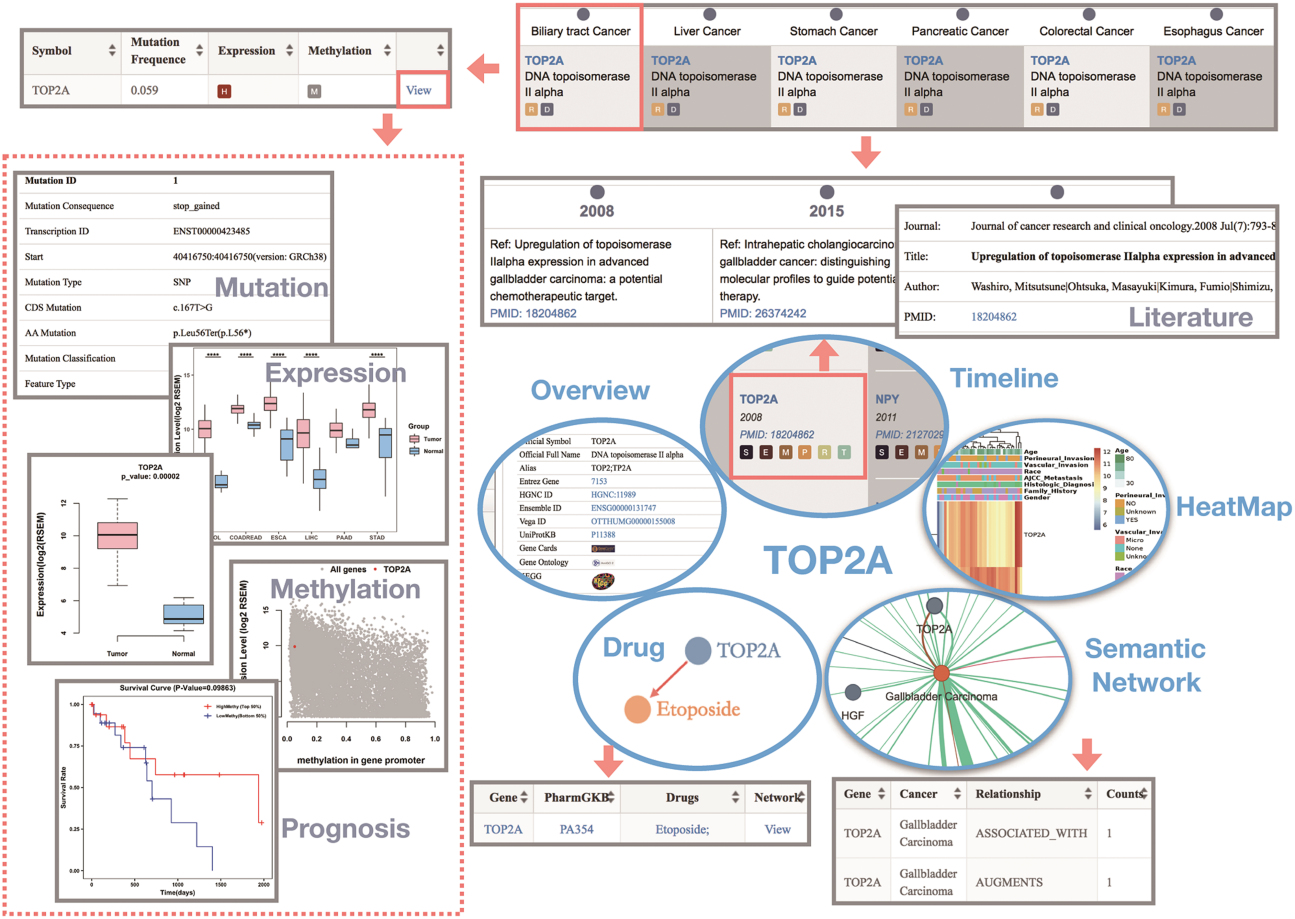
The use of the GIDB with the example of the TOP2A gene in BTC. It shows the query results of each function module related to BTC by searching `TOP2A’ in the GIDB.

Moreover, two missense variants and a stop-gain variant that have a high putative impact on the encoded protein have been annotated. Significantly high expression of TOP2A was found not only in CHOL tumour samples compared with the non-tumour samples but also in COCA, ESCA, LIHC and STAD (all *P* < 0.0001). Among them, DNA methylation levels within the TOP2A promoter region were only significantly lower in LIHC [false discovery rate (FDR) < 0.0001]. The semantic network presents a global view of how the TOP2A gene is involved in BTC. Two semantic relations (ASSOCIATED WITH and ARGUMENTS) were predicted by SemRep, implying that the TOP2A gene might expand or stimulate the BTC process. Furthermore, a TOP2A targeted drug, Etoposide, has been annotated in the dictionary of cancer drugs and genes. However, this agent has not been reported as a form of chemotherapy for BTC (Figure 4).

## Methods

### Gene–GI cancer associations: GIDB text mining

#### Retrieval of citations using the Entrez Programming Utilities

We investigated the relation between the molecular signatures and GI cancers through an automated NLP approach. The PubMed database (http://www.ncbi.nlm.nih.gov/PubMed) is used as a source of biomedical literature resources for text mining. A set of query terms were constructed based on the Medical Subject Headings (MeSH) database and the National Center for Biotechnology Information Entrez Gene database. Entrez Programming Utilities (32) is applied to extract citations in the MEDLINE format. Through November 2018, we have collected over 370 000 citations from PubMed via preliminary screening.

#### Vocabulary construction

Gene symbols, as well as GI cancer names are mapped to the Concept Unique Identifiers based on the UMLS Metathesaurus. UMLS is a large thesaurus that brings together many biomedical vocabularies (e.g. MeSH). With this thesaurus, a list of UMLS concepts in MEDLINE abstracts was identified by a UMLS NLP tool, MetaMap (2016 version) (33). To concentrate on the relationship between molecular signatures and GI cancers, we limited the UMLS concepts to three semantic types: `Gene or Genome’, `Amino Acid, Peptide, or Protein, Enzyme’ and `Neoplastic Process’. In this version of GIDB, we generated a vocabulary of all Entrez gene entries and six main types of GI cancer.

#### Term frequency-inverse document frequency scores and PubTator annotations

It is important to note that a UMLS-based knowledge acquisition methodology is imperfect within the context of text mining. Another popular text-weighting scheme, the term frequency-inverse document frequency (TF-IDF) score (34, 35) and a web-based text-mining tool, PubTator (36), are further integrated in our association recognition. TF-IDF consists of two components (TF and IDF) whose score can be considered to identify citations that are more relevant or important for a gene. The formula is as follows:

1) The term i TF in citation j = term i count in citation j (${\mathrm{n}}_{i,j}$/all term count in citation j (${\sum}_k{n}_{k,j}$):

$$ {tf}_{i,j}=\frac{n_{i,j}}{\sum_k{n}_{k,j}} $$

2) The term i IDF = count of all citations in corpus (|D|)/count of citations which contain term i ([$\Big\{\mathrm{j}:{t}_i\in {d}_j\Big\}$]):

$$ {idf}_i=\log \frac{\mid D\mid }{\left[\left\{\mathrm{j}:{t}_i\in {d}_j\right\}\ \right]} $$

3) The term i TF-IDF in citation j = the term i TF in citation j × the term i IDF:

$$ \mathrm{tf}{idf}_{i,j}={tf}_{i,j}\times {\mathrm{i}\mathrm{df}}_{\mathrm{i}} $$

Before calculating the TF-IDF score, four preprocessing steps including stop words removal, strip whitespace, toLower and stemDocument were performed by the `tm’ R package.

#### Co-occurrence strategies

To recognize the genes involved in GI cancers, GIDB applies two co-occurrence strategies. One is based on a co-occurrence pattern of gene symbols and GI cancer names in one citation. The other is based on a co-occurrence UMLS concept pattern. These two strategies can find a direct and a hidden indirect supported evidence for one gene associated with GI cancers, respectively. Finally, at least two of three methods (MetaMap, TF-IDF, PubTator) must provide a consistent result to determine that the association between gene and tumours is identified.

#### Semantic network predictions

Given that the UMLS concept vocabularies correspond to both genes and GI cancers, a UMLS-based biomedical text processing tool, SemRep (37, 38), is applied to construct a semantic network for predicting the interaction of two entities (genes and GI cancers) via integrating concepts of the knowledge-based (MetaMap entity recognition) and their semantic associations (SPECIALIST Lexicon and Xerox POS tagging). To concentrate on the relationship between molecular signatures and GI cancers, we limited the relations to seven semantic types: AFFECTS, ASSOCIATED_WITH, AUGMENTS, CAUSES, DISRUPTS, PREVENTS and TREATS. A semantic network is defined as G (V, E), where V = [v1, v2, v3, ...., vn] is a set of nodes (concepts) and E = [e1, e2, e3, ...., em] is a set of edges (associations). The weight of edges is assigned to the number of supporting evidence. GIDB also built a network analytics tool based on the `visNetwork’ R package. Researchers can upload their own data and produce the interaction network in networkD3 visualizations.

#### Gene–GI cancer associations: GIDB data mining

The repository in GIDB integrated both literature mining and data mining results to eliminate some errors. TCGA resources have been integrated in the current version of GIDB, which include 1874 samples covering 185 oesophageal cancers (ESCA), 633 colorectal cancers (COADREAD), 377 liver cancers (LIHC), 185 pancreatic cancers (PAAD), 443 stomach cancers (STAD) and 51 bile duct cancers (CHOL). Data on transcriptomic profiling, simple nucleotide variations and DNA methylation as well as patient clinical information were retrieved. Molecular alterations in each type of GI cancer were filtered out via multidimensional analyses at multiple levels.

For processing mutation data, the somatic (open access) MAF files generated by the TCGA MuTect pipeline from all available cases were downloaded. We selected 10 mutation classifications defined in TCGA for further analysis, including missense mutation, silent, frame shift del, nonsense mutation, frame shift ins, splice site, in frame del, in frame ins, translation start site and nonstop mutation. The mutation rate is defined as the total number of mutated samples of a gene over all samples in each cancer is considered to be a high mutation frequency above 1%.

For processing transcriptome profiling data, the LIMMA R package was utilized to calculate the differentially expressed genes between tumour and normal samples. The genes were regarded as differentially expressed when their absolute value of mean log2-fold change ratios were larger than 1, and the FDR adjusted *P* value was less than 0.05.

For processing DNA methylation data, based on Illumina Human Methylation 450 microarray data, the average beta value of probes in the promoter region was used to represent the methylation status of each gene. The promoters were defined as being 1 kb upstream or downstream of the transcriptional start sites. We performed methylation analysis on normal and cancer pairs of tissues based on the method developed by TCGA (39). The genes were regarded as differentially methylated when the absolute value of DNA methylation levels (β value) exceeded 0.1 in tumours compared with that in normal tissue, and the FDR adjusted *P* value was less than 0.05.

Finally, the intersection of genes identified by both text mining and data mining are considered to be `Gene signatures’ for GI cancer. Upon integrating multi-level omics data and literature knowledge, we mined over 130 000 manuscripts, and a total of 8730 genes were related to GI cancer with literature and data supporting evidence.

#### Evaluation of automatically extracted associations

For a more detailed assessment of our text-mining method, the performance of this approach was evaluated in terms of manually curating 790 randomly selected abstracts from the corpus. The recall, precision and *F*-score are used to determine the accuracy of our method and are calculated as follows: Precision =

$${\frac{\begin{array}{c} \mathrm{Correctly}\ \mathrm{Predicted}\ \mathrm{Gene}\hbox{-} \mathrm{GI}\ \mathrm{Cancer}\\ \mathrm{Associations}\ \left(\mathrm{True
\  Positive
(TP)}\right)\ \end{array}}{\begin{array}{c}\mathrm{Totally}\ \mathrm{Predicted}\ \mathrm{Gene}\hbox{-} \mathrm{GI}\ \mathrm{Cancer}\\ \mathrm{Associations}\ \left(\mathrm{TP}+\mathrm{False \ Positive (FP)}\right)\end{array}}}$$

Recall =

$$ {\frac{\begin{array}{c} \mathrm{Correctly}\ \mathrm{Predicted}\ \mathrm{Gene}\hbox{-} \mathrm{GI}\mathrm{Cancer}\\ \mathrm{Associations}\ \left(\mathrm{TP}\right)\end{array}}{\begin{array}{c}\mathrm{Manually}\ \mathrm{Curated}\ \mathrm{Gene}\hbox{-} \mathrm{GI}\ \mathrm{Cancer}\\ \mathrm{Associations}\ \left(\mathrm{TP}+\mathrm{False \ Negative (FN)}\right)\end{array}}} $$

F-Score = 2×

$$\frac{\mathrm{Recall}\times \mathrm{Precision}}{\mathrm{Recall}+\mathrm{Precision}} $$

In addition, a manual curation effort by dedicated experts will provide regular updates and release the information to our database.

## Discussion

The value of various databases and platforms like PED (22), OMIM (24), COSMIC (25), HGMD (26), Oncomine (27) and cBioPortal (28) should not be underestimated. However, there are neither tools nor databases that provide the same information for GI cancer research as GIDB. GIDB is the first study to retrieve detailed supporting evidence on lists of molecular signatures accumulated from the massive amounts of literature and GI cancer genomic datasets. Using GIDB, researchers in the field of GI cancer can have convenient access to obtain an informative, detailed and itemized summary of characteristics of the molecular signatures most related to GI cancers.

GIDB covers different types of information from varied sources. Thanks to this integration, it offers several advantages. First, GIDB provides a number of annotations of molecular signatures and GI cancers and their relationships by means of a semantic network. Second, GIDB clearly describes the `Timeline’ feature, taking into account the historical knowledge of genes across all GI cancer types. Third, GIDB develops online analysis tools that enable researchers to get a quick glimpse of interaction networks or clustering maps. More importantly, GIDB allows researchers to view the abnormal behaviours of curated signatures at the molecular level, based on three intuitive interfaces: (i) the `Timeline’ feature gives an overview of the historical gene research in GI cancer and explores gene characteristics; (ii) the `Primary Site’ interface provides an additional comprehensive insight into the word cloud of genes most relevant to GI cancers and well-curated signatures (gene, miRNA, lncRNA, CNV, fusion gene, network); and (iii) the `Gene’ interface in each type of GI cancer helps researchers to understand the molecular changes of genes to further decide whether or not to perform wet laboratory experiments for in-depth analysis.

Nevertheless, GIDB is still limited in the following ways. First, both co-occurrence strategies can cover a majority of the signatures methodologically. Still, they lead to some recognition errors, which means that a manual check effort is needed. We analysed the evidence citations automatically retrieved and classified them into three categories:

1) Curation: the evidence statement is clear in the citation.
2) Exclusion: the citation is not correctly recognized, which is often the case with multivocal abbreviations and ambiguous aliases.

e.g. HBD gene, hilar bile duct (HBD) or hepatobiliary diseases (HBD)***.***

e.g. C1419208: GOV (RAB3D gene) (Gene or Genome) and the sentence `The statement was made available on the World Wide Web at http://consensus.nih.gov immediately after the conference’.

3) Ambiguous: The evidence statement is uncertain, which is often the case with indirect evidence and review articles.
  e.g. `FasR is a transmembrane glycoprotein, which belongs to the nerve growth factor/tumor necrosis factor (NGF/TNF) receptor superfamily.... Certainly, the coexpression of FasR, FasL, and other PCD-related proteins has also been reported in other human malignancies: breast cancer, colorectal carcinomas, large granular lymphocytic leukaemia of T or NK cell origin, melanomas, lung, prostate, pancreas, and hepatocellular carcinomas.’

In the current version of GIDB, we construct a general string library and integrated data mining to increase the precision for text-mining results. In future work, GIDB will continue to be updated as follows. One way would be through improving the identification of molecular signatures. For instance, a wrong-word library with high-quality concepts could be used to enhance this text-mining approach and reduce the labour. Another way would be to develop a machine learning algorithm. Moreover, new tools will be further developed to increase the flexibility and utility of our database. The associations between genes and tumour subtypes like carcinoma of gallbladder and intrahepatic cholangiocarcinomas will be improved. Additionally, we plan to add more samples by integrating other public resources like GEO (40) and ICGC (41) for in-depth analysis. In the future, we will continuously improve the automated method for reducing our manual work and expand GIDB’s signature collections once a year.

## Conclusion

GIDB is a GI cancer panoptic integrated database containing straightforward interfaces for well-curated signatures and molecular alterations. We believe that GIDB would significantly save time and effort of researchers to collect information about signatures involved in GI cancer, serve as a reference database for genes showing molecular alterations, help in understanding the molecular events underlying the tumours and prioritize potential biomarkers for further in-depth studies and for precision medicine.

## Notes

**Database URL**: http://bmtongji.cn/GIDB/index.html
